# Microglia and macrophages in glioblastoma: landscapes and treatment directions

**DOI:** 10.1002/1878-0261.13657

**Published:** 2024-05-07

**Authors:** Georgios Solomou, Adam M. H. Young, Harry J. C. J. Bulstrode

**Affiliations:** ^1^ Wellcome MRC Cambridge Stem Cell Institute University of Cambridge UK; ^2^ Department of Neurosurgery Addenbrooke's Hospital Cambridge UK

**Keywords:** glioblastoma, glioma, immune, macrophages, microenvironment, microglia

## Abstract

Glioblastoma is the most common primary malignant tumour of the central nervous system and remains uniformly and rapidly fatal. The tumour‐associated macrophage (TAM) compartment comprises brain‐resident microglia and bone marrow‐derived macrophages (BMDMs) recruited from the periphery. Immune‐suppressive and tumour‐supportive TAM cell states predominate in glioblastoma, and immunotherapies, which have achieved striking success in other solid tumours have consistently failed to improve survival in this ‘immune‐cold’ niche context. Hypoxic and necrotic regions in the tumour core are found to enrich, especially in anti‐inflammatory and immune‐suppressive TAM cell states. Microglia predominate at the invasive tumour margin and express pro‐inflammatory and interferon TAM cell signatures. Depletion of TAMs, or repolarisation towards a pro‐inflammatory state, are appealing therapeutic strategies and will depend on effective understanding and classification of TAM cell ontogeny and state based on new single‐cell and spatial multi‐omic in situ profiling. Here, we explore the application of these datasets to expand and refine TAM characterisation, to inform improved modelling approaches, and ultimately underpin the effective manipulation of function.

## Graphical abstract

The glioblastoma microenvironment is dominated by tumour‐associated macrophages (TAMs). In the tumour core, monocyte‐derived TAMs accumulate and exhibit anti‐inflammatory and immune‐suppressive expression profiles. At the periphery of the tumour, brain microglia‐derived TAMs predominate and exhibit pro‐inflammatory and interferon states. Depletion or repolarisation of selected TAM subsets may be key to effective immunotherapy.

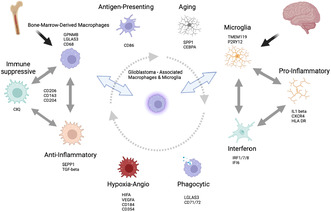

**Abbreviations**
ADAM8: ADAM metallopeptidase domain 8
Angio‐TAMs: pro‐angiogenic TAMs
APOE: apolipoprotein E
APP: amyloid beta precursor protein
AREG: amphiregulin
BBB: blood–brain barrier
BDNF: brain‐derived neurotrophic factor
BIN1: bridging integrator 1
BM: bone marrow
BMDM: bone marrow‐derived macrophages
BNIP3: Bcl‐2 interacting protein 3
C1q: complement component 1q
CAR‐M: chimeric antigen receptor macrophages
CCL2: CC motif chemokine ligand 2
CCR5: c‐c chemokine receptor 5
cGAMP: cyclic GMP‐AMP
cGAS: cyclic GMP‐AMP synthase
CLEC12A: C‐type lectin domain family 12 member A
CNS: central nervous system
COPA: coatomer‐associated protein subunit alpha
CSF1R: colony‐stimulating factor 1
CTLA4: cytotoxic T‐lymphocyte antigen 4
DAMP: damage‐associated molecular patterns
ECM: extracellular matrix
Emilin2: elastin microfibril interfacer 2
EREG: epiregulin
FABP5: fatty acid binding protein 5
FACS: fluorescent antibody cell sorting
FCRLS: Fc receptor‐like molecule
FGF: fibroblast growth factor
FOLR2: folate receptor beta
FPR3: formyl peptide receptor 3
GBP1: guanylate‐binding protein 1
Gda: guanine deaminase
GDNF: glial cell‐derived neurotrophic factor
GEMs: genetically engineered macrophages
GM‐CSF: granulocyte‐macrophage colony‐stimulating factor
GPNMB: glycoprotein non‐metastatic B
HBEGF: heparin‐binding EGF‐like growth factor
HIF‐1α: hypoxia‐inducible factor 1a
Hp: haptoglobin
HSC: haematopoietic stem cell
ICOSLG: inducible T cell costimulator ligand
IFI: interferon alpha inducible
IFIT: interferon‐induced with tetratricopeptide repeats
IFITM: interferon‐induced transmembrane protein
IFN‐TAMs: interferon‐primed TAMs
IFN‐γ: interferon‐gamma
IGF1: insulin‐like growth factor 1
IL: interleukin
IL‐1α: interleukin one alpha
IL‐1β: interleukin one beta
Inflam‐TAMs: inflammatory cytokine‐enriched TAMs
IRF: interferon regulatory factor
ISG: interferon‐stimulated gene
ITGA4: integrin subunit alpha 4
KLRB1: NK cell receptor
KYNU: kynureninase gene
LA‐TAMs: lipid‐associated TAMs
LDL: low‐density lipoprotein
LGALS3: galectin 3
LPL: lipoprotein lipase
LPS: lipopolysaccharide
Ly6C: lymphocyte antigen 6
LYZ: lysozyme gene
MARCKS: myristoylated alanine‐rich C‐kinase substrate
MES‐like: mesenchymal like
MHC‐II: major histocompatibility complex II
MIF: macrophage inhibitory factor
MMDSCs: monocytic myeloid‐derived suppressor cells
MMP: matrix metalloproteinase
MRC1: mannose receptor c‐type 1
MT1: membrane‐type 1
NF1: neurofibromatosis type 1
NFKBI: NF‐kappa‐B transcription factor inhibitor
NGF: nerve growth factor
OSMR: oncostatin M receptor
PAMPs: patho yolk‐sac gen‐associated molecular patterns
PD1: programmed cell death protein 1
PDCD1LG2: programmed cell death 1 ligand 2
PD‐L1: programmed cell death ligand 1
Prolif‐TAMs: proliferating TAMs
Reg‐TAMs: immune regulatory TAMs
RNASE1: ribonuclease 1
RTM‐like TAMs: resident tissue macrophage‐like TAMs
SAll1: spalt‐like transcription factor 1
SCIN: scinderin
scRNA‐seq: single‐cell RNA sequencing
Sell: selectin L
SELPLG: selectin p ligand
SEPP1: selenoprotein P
SLC: solute carrier family
SPP1: osteopontin
STAT3: signal transducer and activator of transcription 3
STING: stimulator of interferon genes
TAM: tumour‐associated macrophages and microglia
TBK1: Tank‐binding kinase I
TGFBI: transforming growth factor beta‐induced
TGF‐β: transforming growth factor beta
TLR4: Toll‐like receptor 4
Tmem 119: transmembrane protein 119
TNC: tenascin C
TNFRSF14: TNF receptor superfamily member 14
TNF‐α: factor‐alpha
TREM2: triggering receptor expressed on myeloid cells‐2
WHO: World Health Organization

## 1 Introduction

Glioblastoma is the most common primary malignant tumour of the central nervous system (CNS) [1, 2]. The World Health Organization (WHO) 2021 Classification of CNS Tumours reserves the glioblastoma descriptor for IDH‐wildtype tumours specifically, reflecting distinct tumour and microenvironment biology compared to other diffuse gliomas such as Astrocytoma IDH‐mutant and Diffuse midline glioma [3, 4]. Standard of care treatment for glioblastoma is typically limited to surgical debulking and chemoradiotherapy. The median survival is 12–16 months, and the 5‐year survival rate is 6.8%. Therefore, the disease is uniformly and rapidly fatal [5]. Strikingly, while many solid tumours exhibit good response to standard treatment, immunotherapy approaches, including cancer vaccines, checkpoint inhibitors and adoptive cell therapies, have failed to achieve marked survival improvements in glioblastoma [6, 7, 8, 9, 10, 11, 12].

The tumour microenvironment plays a central role in glioblastoma immunotherapy resistance, as seen in other ‘immune‐cold’ tumours—for example, in the pancreas, breast, ovary and prostate [13]. In particular, the glioblastoma cellular microenvironment is dominated by tumour‐associated macrophages and microglia (TAMs) [14]. TAMs comprise a mixture of brain‐resident microglia and peripheral monocyte‐derived macrophages, which can outnumber malignant cells in some cases [15, 16, 17]. In contrast, T‐cell infiltration in glioblastoma is typically sparse, with a preponderance of T‐regulatory cells and associated exclusion of CD8^+^ cytotoxic and CD4^+^ helper populations [18, 19]. Importantly, these microenvironment attributes are not well captured in carcinogen‐induced immunocompetent glioblastoma mouse models, which can be highly immunogenic compared to human disease [20]. Therefore, immunotherapy development has significant challenges [21].

Across a range of solid organ cancers, the extent of TAM infiltration predicts poor prognosis. It is associated with malignant proliferation, invasion and immune evasion through tumour and microenvironment cell interactions [22, 23]. In glioblastoma specifically, high TAM cell counts predict poor treatment response and reduced survival [24]. Co‐culture experiments demonstrate TAM‐dependent glioma stem cell (GSC) proliferation [25, 26] and invasion [27, 28]. Mice transplanted with a combination of TAMs and GSCs succumb significantly faster than mice transplanted with GSCs alone [20]. TAMs support glioma stem cell self‐renewal through the secretion of a panoply of growth factors reviewed comprehensively elsewhere [9]. Malignant progression in glioblastoma correlates with and may depend on, TAM cell state transitions [29, 30], and TAM cells can inhibit the treatment response to immune checkpoint blockade [31].

It may be possible to exploit TAM plasticity to disrupt tumour progression rather than promote it. The induction of pro‐inflammatory anti‐tumour states has been associated with recruitment of cytotoxic effector cells and tumour remission [32]. The TAM fraction cannot ‘escape’ therapy through the outgrowth of resistant clones typical in malignant cell populations [33, 34]. Therefore, ablation or repolarisation of the TAM compartment is an attractive treatment possibility for glioblastoma and solid organ cancers in general [35, 36, 37].

Traditionally, the classification of TAM cell states has been based on human macrophage models *in vitro* and rodent models *in vivo*. TAM cell states in these models differ significantly from cell states seen in human tissue, not only in terms of gene expression but also function [38, 39, 40]. Large‐scale single‐cell RNA sequencing (scRNA‐seq) studies in humans demonstrate distinct and diverse human TAM states [41, 42, 43, 44]. Additional complexity specific to glioblastoma stems from the mixed composition of the myeloid cell compartment, comprising separate brain‐resident yolk sac‐derived microglia and bone marrow‐derived macrophage lineages [45, 46, 47].

Understanding the mixture of myeloid cell origins and functional states in human glioblastoma tissue may be key to manipulating this compartment and improving the treatment effect. Parallels with TAM populations in other cancers are instructive. However, even the comparison to brain metastases reveals striking differences in TAM infiltration, ontogeny, and state, even though these tumours share a common pre‐malignant tissue microenvironment [16]. There is also significant TAM diversity in morphology, function, and surface markers across different organs. Regulation of TAM characteristics can be organ and cancer‐specific, particularly by the specifics of the TAM malignant cell interactions in each cancer type [48, 49, 50, 51].

Therefore, a key priority is arriving at a TAM classification for glioblastoma that incorporates biology, function and treatment prediction. Here, we will describe recent progress made towards such a working classification, from studies building on single‐cell and spatial profiling approaches and incorporating cell origin, marker expression and predicted function. We will discuss current TAM‐directed treatment approaches in glioblastoma and explore state‐of‐the‐art models that will enable interrogation and manipulation of the TAM population in the future.

## 2 Myeloid cells in brain and glioblastoma

Microglia were initially described by Rio‐Hortega in 1919 as a leukocyte‐like brain cell population of mesodermal origin. Subsequently, they were the first tissue‐resident macrophage population to be recognised [52]. Lineage‐tracing and fate‐mapping studies in mice indicate that microglia arise from immature myeloid progenitors in the yolk sac [46]. Migrating microglia between embryonic day E8 and E9.5 are responsible for seeding the brain. Subsequent self‐renewal proceeds independent of the circulating bone marrow‐derived monocyte pool. The turnover of microglia is slow, and these cells can persist for decades in the human brain [53]. Transcriptional diversity across brain regions has also been reported, although its functional significance is uncertain [54].

### 2.1 Microglia diversity in development, health and disease

The processes of proliferation and repopulation of microglia occur from within the microglia compartment exclusively in the resting CNS. Broadly, this also occurs following injury, provided the blood–brain barrier (BBB) remains intact [45]. Immediately adjacent to the brain parenchyma, ‘border‐associated’ or ‘CNS‐associated’ macrophages derived from bone marrow occupy a physically distinct niche in choroid plexus, meningeal and perivascular spaces [55]. During embryogenesis, these cells and other bone marrow‐derived macrophages originate from the fetal liver's haematopoietic stem cell (HSC) pool [47, 56]. However, circulating bone marrow‐derived progenitors can also contribute to the repopulation of the parenchyma following CNS damage if BBB integrity is compromised [57, 58]. Following lethal irradiation and bone marrow (BM) transplantation, donor‐derived peripheral monocytes alone can reconstitute this compartment in chimeric mice [59]. Similarly, microglia dominate the microenvironment in the early glioma transformation and infiltration phases. However, malignant progression is associated with hypoxia, necrosis, endothelial cell tight junction breakdown, chemotaxis, and accumulation of peripheral monocyte‐derived macrophages [20]. In parallel, the immune activation signatures characteristic of low‐grade glioma (LGG) are replaced by immunosuppressive expression profiles [30, 44]. For example, in glioma, microglia‐derived TAMs exhibit pro‐inflammatory signatures, whereas most macrophages display immunosuppressive signatures [44, 60]. Additionally, the extent of microglia infiltration correlates with mutation profile and glioma progression. Namely, IDH‐wildtype glioblastomas demonstrate increased macrophage infiltration, whereas IDH mutant gliomas, in particular grade II gliomas, enrich for microglia infiltration [60, 61]. Mutations at the NF1 gene are also associated with increased macrophage infiltration and total TAM numbers [62]. However, whether these observations represent an intrinsic difference based on myeloid cell origin is unclear. It also remains to be determined if such effects could result from differential enrichment of macrophages in the BBB‐deficient tumour core and microglia in the invasive periphery.

### 2.2 Gender differences and myeloid‐derived suppressor cells in glioblastoma

Sex differences in microglia have been observed in the developing and adult brains [63]. For example, microglia from females expressed higher levels of interferon regulatory factor 3 [64, 65], whereas microglia from males exhibited elevated MHC II expression [66, 67, 68]. Differences in expression signatures were preserved in microglia from females transplanted into male mice, pointing to a lasting impact of early hormonal influences. Significant sex differences have also been identified in myeloid‐derived suppressor cells (MDSCs). MDSCs represent a specific TAM subgroup derived from immature circulating monocytes and distinguished by the following features: (a) a downregulation of MHC Class II expression [69], and (b) an upregulation of transcription factors hypoxia‐inducible factor 1a (HIF‐1α) and signal transducer activator of transcription 3 (STAT3), compared to their parent cells [70, 71, 72]. MDSCs potently suppress B cell, NK cell and especially T cell activation across various cancers, including glioblastoma [73, 74, 75]. Selective enrichment of MMDSCs within GBM tumours has been demonstrated in male mice and male patients. In contrast, a separate granulocytic or polymorphonuclear myeloid‐derived suppressor cell (GMDSC or PMN‐MDSC) population is enriched in the bloodstream of GBM‐bearing females [74]. Since host immunosurveillance is key to regulating tumour initiation and progression, sexual dimorphism in microglia, MDSC and TAM compartments may contribute to the established finding of a higher GBM incidence and poorer prognosis in men.

### 2.3 Microglia and macrophage ontogeny and cell surface markers

It is unclear to what extent the role of yolk sac‐derived, brain‐resident microglia is interchangeable with bone marrow‐derived macrophages for tumour progression. However, microglia‐specific biology is important functionally. For example, microglia exhibit distinct electrophysiology compared to macrophages, mediated through potassium channel expression [76, 77]. Spalt‐like transcription factor 1 (SAll1), Pu.1 and interferon regulator factor 8 (Irf8) are selectively expressed by microglia [48, 78, 79]. Microglia‐specific surface antigens common to mice and humans include a transmembrane protein of unknown significance, TMEM119, and the purinergic receptor P2YR12 [80, 81]. A recent meta‐analysis of five previously published murine transcriptional datasets additionally identified solute carrier family two members (Slc2a5) and fc receptor‐like molecule (Fcrls) to be consistently upregulated in microglia. In contrast, elastin microfibril interfacer 2 (*Emilin2*), guanine deaminase (*GDA*), *haptoglobin* (*Hp*) and selectin L (*Sell*) were enriched in macrophages/monocytes in RCAS and GL261 mouse models of glioblastoma [49].

Microglia have also been isolated from macrophages using specific fluorescent antibody cell sorting (FACS) marker combinations, such as gating for the CD11b^+^, CD45‐low, CX3 motif chemokine receptor 1 (CX3CR1^+^) microglia population [38]. A CD11b^+^/CD206low/CD163^−^ human microglia population has been distinguished from perivascular macrophages (CD11b^+^/CD206high/CD163^+^) [20, 60, 61, 82, 83]. Moreover, monocyte‐derived TAMs show a high expression of CD163/CD206/CD9 [20, 43, 60, 61, 82, 83, 84, 85] and an upregulation of genes related to growth factor activation (EREG, ligand for EGFR, IFITM2) and folate synthesis (FPR3) and in line with this, the S100A gene family having a range of inflammatory functions [86]. However, caveats apply to marker combinations across species. For example, CX3CR1 expression is restricted to microglia from mice but is common to microglia and monocyte‐derived macrophages from human microglia [61, 87]. Furthermore, lymphocyte antigen 6 (Ly6C) is expressed in mice but not humans [88].

A healthy human consensus ‘core microglia’ gene expression signature would include TMEM119, P2RY12, and CX3CR1. Other reported markers include P2RY13 and selectin p ligand (SELPLG) [43, 67, 68, 85], colony‐stimulating factor (CSF1R), triggering receptor expressed on myeloid cells‐2 (TREM2), c‐c chemokine receptor 5 (CCR5) and Myristoylated alanine‐rich C‐kinase substrate (MARCKS) [20, 43, 61, 80, 83, 84, 85] (Table 2).

Conversely, genes enriched in bone marrow‐derived macrophages (BMDMs) include transforming growth factor beta‐induced (TGFB)I, C‐type lectin domain family 12 member A (CLEC12A), interferon‐induced transmembrane protein 2 (IFITM2), formyl peptide receptor 3 (FPR3), S100A11, kynureninase (KYNU), integrin subunit alpha 4 (ITGA4) [89], monocyte derivation surface markers CD184 and CD354 [20]. There is significant variability across data sets [84, 90], and a (non‐exhaustive) summary of differential key human glioma‐associated macrophages and microglia marker expression is provided in Table 1.

**Table 1:** Key surface markers for attribution of ontogeny and cell state across mixed tumour associated macrophage and microglia populations.

Marker	MG	MΦ	Comment	References
CCR2		+	CCL2/CCR2 ligand‐receptor pair contributes to monocyte infiltration	[98, 101, 102]
CLEC12A		+	Myeloid inhibitory receptor regulates inflammation negatively	[61, 70, 105]
CX3CR1	++	+	Chemokine receptor associated with activation and migration	[67, 72, 73, 87]
HLA DR	+	+	MHC class II antigen presentation component Upregulated in response to tissue damage	[73, 91]
IBA1	+	+	Calcium‐binding protein upregulated on activation	[61, 73, 82, 83]
P2RY12	++		Purinergic receptor involved in motility and migration Downregulated on activation	[70, 73, 74, 85]
SALL1	+		Specific to microglia and associated with resting state Downregulation drives inflammatory phenotype and phagocytosis	[90]
SIRPa	+	+	Receptor of CD47. CD47‐SIRPα axis conducts the process of anti‐phagocytic, referencing to “don't eat me” signal	[103]
Siglec‐10		+	Receptor of CD24. CD24–Siglec‐10 signalling regulate phagocytic effects	[104]
SPP1	+		Also called Osteopontin Cytokine upregulated in disease‐associated microglia	[69, 73, 88, 89]
TGFBI	+	+	Growth factor receptor shown to affect immunogenicity, alter polarisation and dampen the inflammatory phenotype	[61, 70, 105]
TMEM119	++		Resting human and murine microglia but not macrophages. Downregulated on activation	[43, 67, 84, 86]
CD11b	+	+	Pan‐myeloid lineage marker	[70, 74, 84]
CD14	+	+	Immunosuppressive phenotypes Expressed on the membrane of mature myeloid cells	[67, 69, 96]
CD36		+	Scavenger receptor (CD36) Markers of monocyte‐to‐macrophage transition	[69, 74, 91]
CD68	+	+	Glycoprotein expression associated with phagocytosis. Pan‐macrophage surface receptor	[61, 87, 93, 94, 95]
CD69	+		Microglia cell surface marker Immune regulation and T‐cell activation/suppression	[70, 97]
CD71/72		+	Monocyte‐derived glioma macrophages with phagocytic function	[70]
CD74	+		MHC class II antigen presentation component Receptor binding drives pro‐inflammatory phenotypes	[73, 91, 92]
CD86	+	+	Antigen presentation	[69, 70, 71, 72, 73]
CD163/206		++	Infiltrating CNS macrophages Immunosuppressive states, (CD 206) antigen presentation and phagocytosis	[70, 74, 91, 98, 99, 100]
CD 184/354		+	Microglia and macrophage markers. Hypoxic signature	[70]

## 3 TAM states *in vitro* – M0/M1/M2

Concepts of macrophage activation state date to the 1960s, when the ‘activated’ descriptor was first applied to populations primed for antimicrobial and anti‐tumour activity [91, 92]. In the brain specifically, the transition of microglia from a resting to an activated state is associated with a striking morphological transition from ramified to amoeboid. The ‘classically‐activated’ or ‘M1’ phenotype is induced by toll‐like receptor binding of damage‐associated molecular patterns (DAMPs) and pathogen‐associated molecular patterns (PAMPs). Exposure of ‘undifferentiated’/‘resting’/‘M0’ macrophages to Toll‐like receptor 4 (TLR4) ligands, interferon‐gamma (IFN‐γ), lipopolysaccharide (LPS) or granulocyte‐macrophage colony‐stimulating factor (GM‐CSF) can induce the M1 state *in vitro* [93, 94, 95]. The M1 phenotype has been attributed pro‐inflammatory and tumour‐suppressive activity, including enhanced antigen presentation through expression of MHC class II [93, 94, 95] and immune cell recruitment through the production of cytokines including tumour necrosis factor‐alpha (TNF‐α), interleukin one beta (IL‐1β), interleukin one alpha (IL‐1α), complement component 1q (C1q), interleukin 6 (IL‐6), interleukin 12 (IL‐12), and CC motif chemokine ligand 2 (CCL2) [96]. Expression of nuclear transcription factors such as NF‐kappa‐B transcription factor (NFkB) – NFkBinhibitor (NFKBI) Z, NFKBIA, and an interferon‐stimulated gene signature are typical hallmarks of the M1‐like phenotype activation state [interferon‐induced with tetratricopeptide repeats (IFITM) 3, IFIT1, Interferon alpha inducible (IFI) 6, interferon‐stimulated gene (ISG) 15/20] [20, 60, 61, 82, 83].

The ‘alternatively‐activated’ M2 activation state is broadly considered anti‐inflammatory and can be induced by exposure to IL‐4, IL‐10 and IL‐13 [97, 98]. The M2 state is associated with the production of anti‐inflammatory mediators including ARG1, IL‐10, and IL‐4, and of pro‐tumoural growth and neurotrophic factors including transforming growth factor beta (TGF‐β), fibroblast growth factor (FGF), CSF1, insulin‐like growth factor 1 (IGF1), brain‐derived neurotrophic factor (BDNF), nerve growth factor (NGF), neurotrophins, and glial cell‐derived neurotrophic factor (GDNF) [93, 94, 95]. M2 cells also produce tissue remodelling and angiogenic factors, including vascular endothelial growth factor (VEGF), matrix metalloproteinase (MMP) 2, MMP9 and membrane‐type 1 (MT1) – MMP [17, 97, 99]. Characteristic surface markers include CD163, CD9, CD14, CD204/MSR1, and CD206 [20, 60, 61, 82, 83].

The ‘M0/M1/M2’ classification offers a functional annotation of cell activation states, which can be attained in defined conditions *in vitro* [100]. These macrophage cell states have been defined by analogy to the type 1 T‐helper (Th1) and type 2 T‐helper (Th2) cell states. The terms ‘M1‐like’ and ‘M2‐like’ have been applied to build on these key functional distinctions by extending the annotation to *in vivo* macrophage and TAM populations in glioblastoma and other cancer contexts. Many tumour‐expressed factors are associated with the induction of ‘M2’ activation states, and traditionally, tumour‐associated macrophages have been considered an M2‐like population [98]. Further subclassification of the ‘M2 state’ has been proposed [97, 98, 99, 101] to incorporate i) a pro‐invasive IL‐4/IL‐13 driven ‘M2a’ subset, an IL‐1R ligand, an LPS‐driven ‘M2b’ subset, and an IL‐10 and TGF‐β driven ‘M2c’ subset promoting angiogenesis [102, 103]. However, whereas the glioblastoma tumour core has been found to enrich macrophage‐derived TAMs adopting ‘M2‐like’ states, the invasive tumour margins are rich in microglia‐derived TAMs and ‘M1‐like’ expression patterns [104]. Some key glioma cell surface and secreted factors also seem to induce ‘M1‐like’ rather than ‘M2‐like’ states [105, 106]. Although bulk profiling and binary classification cannot fully capture the complexity of patient tumours, the M0/M1/M2 distinction continues to find echoes in the pro‐inflammatory vs immunosuppressive states spectrum emerging from the latest single‐cell transcriptomic datasets [44]. Individual markers previously attributed to *in vitro* phenotypes are still key to understanding function (Table 2).

**Table 2:** Glioma‐associated microglia and macrophage signature genes and cell surface markers by ontogeny and state including, key transcription factors (bold type).

Descriptive	Cluster signature genes	Cell surface markers	References
Microglia (healthy)	*P2YR12*, *TMEM119*, *CX3CR1*, *SELPLG*, *P2RY13*, *CSF1R*, *TGF‐beta 1*, *CCR5*, ***PU.1*** *EMR1*, *TREM2*, *SLC2A5*, *MARCKS*	CD11b, CD32, CD64, CD91, CD 115, CD172a	[73–76]
Tumour‐associated microglia	*P2RY12*, *TMEM119*, *CX3CR1 (core)* *NAV3*, *SINGLEC8*, *SLC1A3*, *APOE*, *LPL*, *IFI27*, *IFITM3*, *VEGFA*, *SORL1*, *SPRY1*, *SRGAP28*, *BIN1*, *SCIN*, *DUSPI*, *FOS*, *TFRC. EGR3*, *TREM1*, *LMNA*, *RGS1*, *PLEK*, ***BHLHE41*, *HIF1A***	CD69, CD83, CD151, CD163	[69–73]
Bone marrow‐derived tumour‐associated macrophages	*S100A1*, *GFBI*, *IFITM2*, *FPR3*, *KYNU ITGA4*, *EREG*, *S100A6*, *LYZ*, *GPNMB*, *LGALS3*, *FCN1*, *VCAN*, *FLNA*, *CCR2*, *CTSD*	CD9, CD36, CD44, CD49D, CD63, CD65, CD68, CD163, CD206, CD209	[69–76]
Anti‐inflammatory/immunosuppressive	*SEPP1*, *SLC40A1*, *FOLR2*, *MRC1*, *RNASE1*, *CCL18*, *CCL13*, *LGMN*, *STAB1*, *PLA2G7*, *IL2RA*, *FN1*, *MARCO*, *NAMPT*, *FOSL2*, *TGFBI*, *S100A4*, *LYZ*, *VEGFA*, *IL10*, *LYVE1*, *COLEC12*, *CTSB*, *NRP1*	CD9, CD14, CD16, CD163, CD204, CD206	[69–73]
Pro‐inflammatory	*HLA‐DRA*, *IL23A*, *CCL20*, *CCR7*, *CXCL8*, *IL1RN*, *CCL4L2*, *CCL3*, *IL6*, *CCL3/MIP‐a*, *CCL4/MIP‐β*, *CCL3L3*, *CCL4L2*, *TNF*, *IL1beta*, *CCL2*, *IL1A*, *OSM*, *PDK4*, *CXCR4*, *RHOB*, ***NFKBIA*, *NFKBIZ***	CD83	[69–73]
Hypoxic/angiogenic	*BNIP3*, *ADAM8*, *MIF*, *SLC2A1*, *LDHA*, *ERO1A*, *HILPDA*, *HK2*, *VEGFA*, *ENO1*, *P4HB*, ***HIFA***	CD184, CD354	[69–73]
Interferon	*CXCL10*, *CXCL11*, *GBP1*, *LY6E*, *IFI6*, *ISG15*, *IFITM3*, *CXCL9*, *IFIT1*, *IDO1*, *ISG20*, *CCL2*, *IFI27*, *MX1*, ***IRF1/7*, *STAT1***	N/A	[69–73]
Homeostatic/naive	*S100A4*, *LGALS1*, *NRG1*, *VCAN*, *VEGFA*, *FCN1*, *LYZ*, *TIMP1*, *CST3*	CD44, CD52	[69–73]
Antigen‐presenting	*CX3CR1*, *IFNGR1*, *TGFB1*, *B2M*	CD86	[69–73]
Proliferative	*MKi67*, *TOP2A*, *CENPF*, *CCNA2*, *CDK1*	N/A	[69–73]
Phagocytic/lipid	*GPNMB*, *LGALS3*, *FABP5*	CD71, CD72	[69–73]
Ageing	*SPP1*, *APOE/C*, *BIN1*, *PLCG2*, *APOG1*, *TMIGD3*, *SLP1*, *PHKG1*, ***CEBPA***	N/A	[69–73]

## 4 New single‐cell and spatial perspectives

The myeloid cell transcriptome, epigenome and even proteome are now accessible in full or across broad gene panels, at or close to single‐cell resolution. These datasets have significantly expanded and refined our understanding of the myeloid compartment in the normal human brain, across disease states, and even in response to treatment [20, 43, 60, 61, 82, 83, 107, 108, 109]. Several themes emerging from these efforts promise to inform improved functional classification in the future.

### 4.1 Microlgia and macrophages retain a signature of origin and acquire niche‐specific spatially‐differentiated markers

**Fig. 1:**
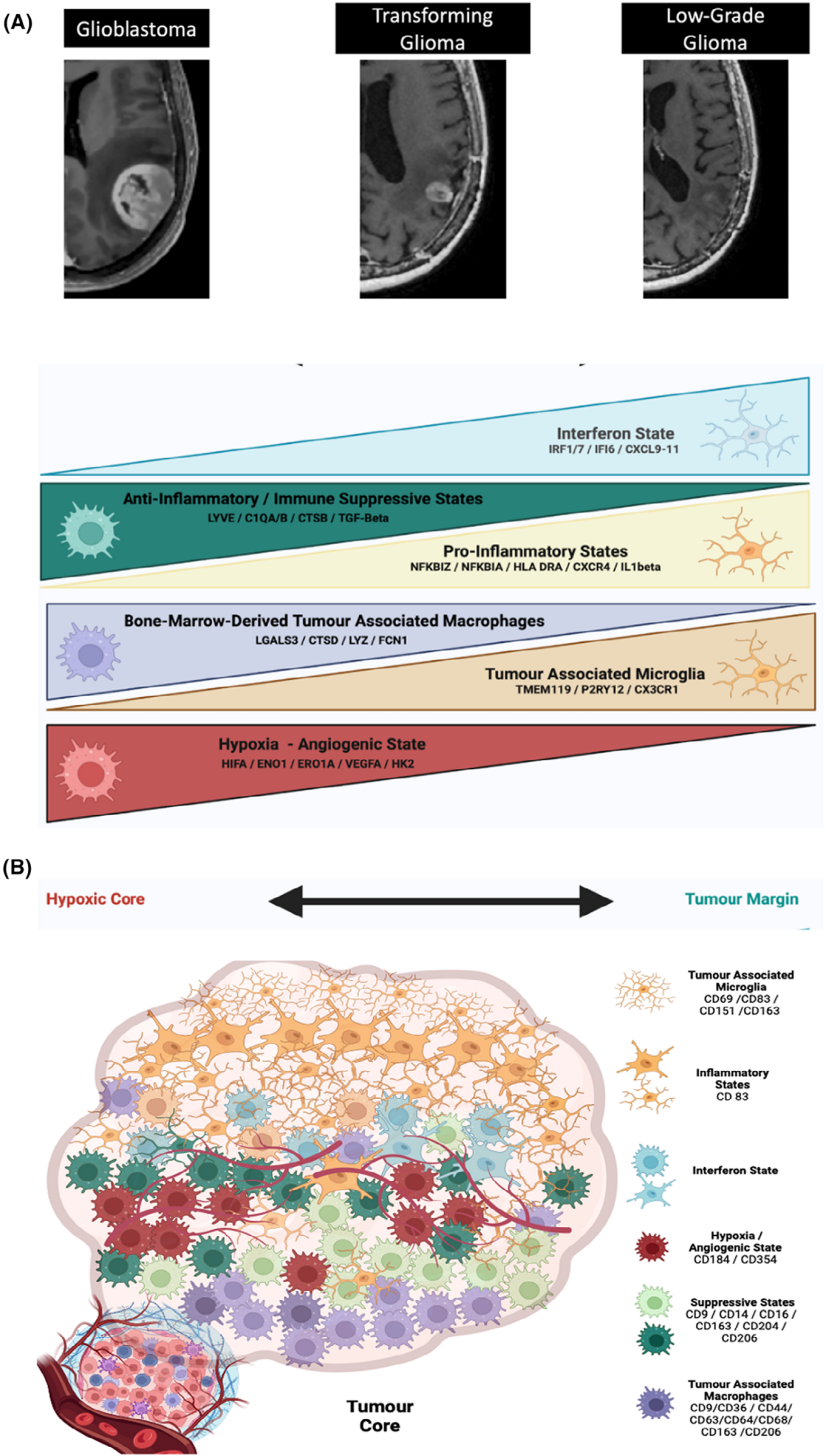
Typical TAM cell ontogeny and state enrichments throughout malignant progression (A) and by spatial location in the tumour (B).

Unsupervised clustering of single‐cell transcriptomic profiles distinguishes brain‐resident microglia and circulating monocytes/macrophages, and spatial transcriptomics confirms the enrichment of these subpopulations in distinct niches within each glioblastoma sample [20, 44, 61, 82, 110] (Table 2). In particular, glioma‐associated microglia are enriched at the invading tumour margin [20, 60, 61, 82, 83, 109], whereas monocyte‐derived macrophages are located preferentially at the tumour core (Fig. 1A,B). Here, dense cellular packing and poor vascularity result in low oxygen tension, HIF stabilisation and hypoxic/angiogenic TAM states associated with angiogenic growth factor secretion [89, 111, 112].

Although signatures of cell origin are retained, peripheral macrophages are nevertheless found to upregulate microglia‐associated markers such us TMEM119 and P2RY12 on exposure to the brain microenvironment. Conversely, microglia can acquire monocyte/macrophage profiles in response to blood–brain barrier breakdown for example CD206 and CD163 [16, 44].

Single‐cell profiling has confirmed and elaborated the core human microglia homeostatic gene expression signature – Namely, purinergic receptors P2RY12/13 and TMEM119/SELPG ‘sensosome’ genes previously involved in microglia process extension and sensing were detected via single‐cell profiling. Additionally, cytokines/chemokines TGF‐beta1, CX3CR1, CSF1R and CCR5 mediating cell recruitment and maintenance were also found in these studies [83, 113]. Based on microglia in the ‘normal’ brain (neurosurgical access cortex), Sankowski et al. performed gene ontology (GO) enrichment analysis based on cluster‐enriched gene sets identifying clusters 2 and 3 expressing high levels of core microglia genes CX3CR1 and TMEM119. In contrast, clusters 2, 6 and 7 were characterised by the strong expression of major histocompatibility complex II (MHC‐II) and antiviral immunity genes, such as HLA‐DRA, CD74 and IFI44L [83]. C2 and C3 accounted for > 50% of microglia in their own sample set and previously published normal human datasets [85]. Clusters C6 and C7 expressed integrin receptor protein binding and metabolism genes, osteopontin (SPP1), apolipoprotein E (APOE) and low‐density lipoprotein (LDL), and low levels of CX3CR1. ‘Pro‐inflammatory’ clusters C1, C5, C8 and C9 expressed chemokines and cytokines including CCL2 and IL1beta. In comparison, glioma microglia were found to downregulate the microglia core signature in favour of the disease‐associated microglia (DAM) signature, which is typical of ageing microglia and microglia in neurodegenerative disease [113, 114]. Microglia in pro‐inflammatory clusters were characterised by upregulation of interferon signalling (IFI27, IFITM3), lipo and apo – lipoprotein processing (lipoprotein lipase (LPL), APOE), angiogenesis (VEGFA), immunosuppression and anti‐inflammatory action (TREM 1/2) inflammatory, metabolic, interferon‐ and hypoxia‐associated modules (HLA‐DR, SPP1, TREM2, CD163, APOE, lipopolysaccharide (LPL, IFI27 and IFITM3)) [108]. The term ‘inflamm‐ageing’ has been used to describe parallel responses in macrophage populations [115]. Microglia and macrophages enrich the invasive margins and the tumour core, respectively, retain a signature of origin and upregulate ‘disease‐associated microglia’ and tumour‐niche‐specific markers.

### 4.2 TAM cell activation states in glioblastoma echo TAM subtypes identified in other cancers

Even before the widespread application of single‐cell approaches, it was apparent that the M0/M1/M2 classification could not adequately capture the spectrum of *in vivo* functional states common to myeloid cells across diverse healthy and diseased tissues [116]. Accordingly, in human glioblastoma, individual TAMs typically express markers classically associated with resting ‘M0’ and activated ‘M1’ and ‘M2’ states in combination [61, 103, 117]. Indeed, of nine myeloid cell subtypes distinguished by unsupervised clustering of 83 479 glioblastoma TAM cells, none showed a clear enrichment for reference‐based cell type gene meta modules corresponding to M1 or M2 states, including M2a/b/c/d subtypes [60]. Direct comparisons of glioma‐derived and control microglia in mice likewise identify distinct tumour‐associated states associated with unique marker expression profiles, falling outside the M1/M2 spectrum [118].

A recent classification has incorporated scRNA‐seq and CITE‐Seq profiling to identify six distinct TAM cell clusters [20]. Cluster 1 included ‘transitory monocyte‐derived TAMs’ expressing monocyte genes EREG, S100A6 and lysozyme (LYZ) at high levels, with corresponding downregulation of mature macrophage markers C1QA and IGF1 [20]. Cluster 2 exhibited a phagocytic and lipid metabolism signature, including upregulation of Glycoprotein non‐metastatic B (GPNMB), galectin 3 (LGALS3) and Fatty Acid Binding Protein 5 (FABP5). Cluster 3 exhibited a hypoxic and glycolytic gene expression profile, enriching for Bcl‐2 interacting protein 3 (BNIP3), ADAM metallopeptidase domain 8 (ADAM8), macrophage inhibitory factor (MIF) and Solute Carrier Family (SLC) 2A1. Cluster 4 expressed microglia signature genes, such as CX3CR1, Bridging Integrator 1 (BIN1) and scinderin (SCIN). In contrast, cluster 5 downregulated these genes in favour of an anti‐inflammatory signature, including selenoprotein P (SEPP1), SLC40A1, folate receptor beta (FOLR2), mannose receptor c‐type 1 (MRC1) and ribonuclease 1 (RNASE1) expression. Cluster 6 was associated with interferon‐gamma signatures, including CXCL10, CXCL11, and guanylate‐binding protein 1 (GBP1). Using Cellular Indexing of Transcriptomes and Epitopes by Sequencing (CITE‐seq) and extensive antibody panel broad protein validation, a ‘transitionary’ monocyte‐to‐macrophage subset was captured using CD36 and CD64. Monocyte‐derived (Mo) TAMs captured included CD44/CD49d/CD9 versus CD69/CD151/TMEM119 for microglia‐derived TAMs. SEPP1‐high monocyte‐derived cluster TAMs expressed folate receptor‐β, CD206 and CD209; hypoxic Mo‐TAMs CD184 and CD354; and phagocytic/lipid Mo‐TAMs CD71 and CD72.

Although the microglia TAM subpopulations in the CNS are transcriptionally unique [22, 41], there is a strong correlation between glioblastoma myeloid cell subtypes and pan‐cancer TAM subtypes [22]. This is an encouraging finding since incorporating data and clustering from glioblastoma studies into the wider landscape of TAM states across diverse cancers could enable us to generalise across tumours and accelerate treatment advances. Across a range of cancers, a series of consensus TAM subtypes have been identified by multiple groups: interferon‐primed TAMs (IFN‐TAMs), immune regulatory TAMs (Reg‐TAMs), inflammatory cytokine‐enriched TAMs (Inflam‐TAMs), lipid‐associated TAMs (LA‐TAMs), pro‐angiogenic TAMs (Angio‐TAMs), RTM‐like TAMs (RTM‐TAMs), and proliferating TAMs (Prolif‐TAMs) [22, 41]. Table 2 focuses on emerging glioblastoma myeloid cell functional states, ontogeny, and their distribution within this working classification. Sex‐based differences have also been linked to better male immunotherapy responses [67, 119]. Seeking commonalities across pan‐cancer datasets may be key to understanding this compartment and developing tissue‐agnostic immunomodulatory treatments with selectivity for the TAM compartment over the broader myeloid population. This will be an important consideration to ensure safe application in patients.

Perhaps most promising in functional application, recent work from the Bernstein group has collapsed myeloid cell identity in glioblastoma into two dimensions, echoing a conceptually appealing and widely adopted classification of glioblastoma malignant cell identity [44, 120]. This classification distinguishes two inflammatory and two immunosuppressive myeloid cell subtypes in glioblastoma. The pan‐inflammatory myeloid signature includes CCL3 and CCL4 expression, and the population is further subdivided according to the expression of ‘CXCR4 inflammatory’ (CXCR4, CXCL12, CX3CR1) and ‘IL‐1B inflammatory’ (IL1B, IL1A, CC2, TNF, OSM and CXCL8) signatures. The pan‐immunosuppressive signature includes expression of CD163, and ‘C1Q immunosuppressive’ (C1QA, C1QB, C1QC, CD16, C3, C2, VSIG4) and ‘Scavenger immunosuppressive’ (MRC1, MSR1, LYVE1, COLEC12, and STAB1) are distinguished. Although the cell of origin is not explicit within this classification, microglia and macrophages are shown to express predominantly inflammatory and immunosuppressive signatures, respectively, in keeping with previous reports (Table 2, Fig. 1B).

### 4.3 Malignant cell genetics and expression profile contribute to mutual glioma cell/TAM cell interactions mediating TAM chemotaxis and polarisation

Myeloid cell profiles differ between IDH mutant and IDH mutant WHO grade 4 gliomas at the bulk tumour level, with the former enriching for microglia in inflammatory states and the latter for macrophages in suppressive states. Interestingly, however, myeloid composition may be better predicted by histopathological tumour grade rather than by mutation profile [44]. Neurofibromatosis type 1 (NF1) mutation predicts the extent of TAM infiltration [62], and malignant cell transcription profiles may play a key role. Irrespective of mutation profile, glioblastoma cells converge and depend on conserved neural stem cell and glial progenitor transcriptional identities normally responsible for expansion and proliferation in embryonic development [107, 120, 121, 122, 123]. These identities exist in dynamic equilibrium with each other, adapting to tissue and treatment context [124]. However, an additional ‘mesenchymal’ or ‘injury response’ signature can also be discerned, and this has no direct developmental correlation [120, 125].

Serial mouse glioma transplants in an immune‐competent host expand mesenchymal malignant cell (MES‐like) and TAM fractions, mimicking that seen in recurrent human disease [126]. This process, termed epigenetic immunoediting, was shown to reflect the outgrowth of those malignant cell clones which had succeeded in recruiting immunosuppressive TAMs and escaping adaptive immune clearance through the derepression of myeloid master regulator Irf8.

The resulting signature from epigenetic immunoediting includes the expression of chemokines and interferons responsible for TAM chemotaxis. Conversely, TAMs have been shown to drive glioblastoma cells to a MES‐like state *in vivo* through the STAT3 activation downstream of receptor tyrosine kinase AXL and oncostatin M receptor (OSMR), targeted by Amphiregulin (AREG), Heparin‐binding EGF‐like growth factor (HBEGF) and OSM ligands expressed on macrophages. Accordingly, macrophage depletion therapy reduced MES‐like malignant fraction [127].

New spatial transcriptomic approaches promise to add detail to the landscape of interactions between TAMs, tumour cells, and other components of the tumour microenvironment. Examples of established interactions include the osteopontin pathway (e.g., SPP1‐CD44), which promotes TAM invasion, pro‐tumour polarisation, and stem cell maintenance around blood vessels [128, 129]. Likewise, PTPRZ1 glioma cell/PTN myeloid cell interactions play a key role [130]. TAMs interact with the extracellular matrix (ECM) via tenascin C (TNC) through TAM CD74 receptor binding to MIF, coatomer‐associated protein subunit alpha (COPA), or the Amyloid Beta Precursor Protein (APP) ligand [60]. These signalling networks may be key to establishing and sustaining the MES‐like glioblastoma phenotype.

Tumour‐associated macrophage interactions with the adaptive immune system are likely critical to function. For example, the NK cell receptor (KLRB1) encoding CD161 has been identified as an inhibitory receptor in myeloid cells, mediating reduced T‐cell cytotoxicity and cytokine secretion [131]. Additional interactions of key potential significance have yet to be functionally validated. For example, Darmanis et al. have identified immune checkpoint ligand‐receptor interactions between tumour and myeloid cells. These include but not limited to the programmed cell death Protein 1 (PD1) (CD274 [PDL1], programmed cell death one ligand 2 (PDCD1LG2) [PDL2]) and Cytotoxic T‐Lymphocyte Antigen 4 (CTLA4) (CD80 and CD86), as well as the genes inducible T‐cell costimulator ligand (ICOSLG) (ligand of ICOS receptor), CD276 (B7‐H3), TNF receptor superfamily member 14 (TNFRSF14) (ligand of BTLA), and LGALS9 (ligand of TIM3) [108].

## 5 Modelling TAM function

Currently, rodent models remain the gold standard for modelling the TAM compartment. However, gene expression profiles differ substantially between mice and humans [132], as might be expected given the vast differences in brain function and scale. Additionally, the brain TAM compartment has often been profiled *in vivo* using carcinogen‐induced mouse models, which diverge from patient tumour biology and immune microenvironment. Carcinogen‐induced models present a rich complement of neoantigens for adaptive immune recognition, driving potent responses to immunotherapy, which have subsequently failed to translate in patients whose tumours are much less immunogenic [21].

To date, it has proved difficult to model the TAM compartment faithfully *in vitro* due to the rapid adoption of artefactual cell states in response to removing the normal brain microenvironment [40, 133]. In particular, the microglia core signature, including P2RY12 and TMEM119, is rapidly extinguished in rodent primary microglia at the time of isolation and is not restored even after the acute inflammatory response to dissociation sites [38, 133]. However, recent work demonstrates that human microglia can retain marker expression more convincingly [134]. Certainly, *in vivo*, cell states are not well replicated in immortalised human microglia lines [135, 136]. Similarly, iPS‐derived microglia‐like populations fail to adopt the desired expression profiles outside the brain microenvironment [137]. Whereas better approximation to human brain tissue microglia states was reported in microglia infiltrating organoids *in vitro*, a further xenotransplantation step into a mouse host has been reported to achieve a closer match [137]. This represents a challenging multi‐step process which would not be practical for routine investigation.

There is, therefore, a pressing unmet need for practical tractable *in vitro* models of human TAM cellular identity and function. The goal of capturing representative TAM cell states in primary patient‐derived or iPS‐derived cultures will depend on an improved understanding of the cues in determining cell state. Modelling pairwise malignant cell/TAM cell interactions in glioblastoma has been attempted by several groups [20, 29, 60, 61, 82, 83, 110, 127, 138], and this area presents exciting possibilities for future development.

## 6 Targeting TAMs for treatment

Tumour‐associated macrophages present a conceptually appealing treatment target in glioblastoma as in many other solid organ tumours [139]. In broad terms, the goal is either to deplete or ‘re‐educate’ the TAM compartment globally. Another option is to target specific TAM‐regulated pathways involved in tumour growth signalling, immune suppression, angiogenesis and invasion. Individual treatment strategies can address more than one of these aspects. For example, the CSF1 receptor, expressed exclusively on myeloid/monocytic lineages, is a key driver of macrophage differentiation and is required for survival *in vitro* [140]. CSF1R signalling also plays a role in chemotaxis and recruitment [141]. However, CSF1R blockade with BZL945 in a mouse *in vivo* glioblastoma model resulted in TAM re‐education rather than ablation [35, 142]. In preclinical models, CSF1R inhibitors have demonstrated survival benefits in isolation and combination with IGF‐1 blockade [143], VEGFR2 blockade [144] or radiotherapy [145]. However, these combinations have yet to be validated in human patients. At the same time, monotherapy with the blood–brain barrier penetrant small molecule CSF1R inhibitor Pexidartinib failed to demonstrate an overall survival benefit in recurrent glioblastoma as a monotherapy [146].

### 6.1 TAM compartment depletion

Tumour‐associated macrophage depletion can be achieved by preventing recruitment and retention. Besides CSF1/CSF1R signalling, a blockade of several pathways mediating TAM chemotaxis was attempted. Kynurenine produced by glioma stem cells activates the aryl hydrocarbon receptor on macrophages, resulting in CCR2 upregulation, TAM recruitment and expression of CD39 and CD8^+^ T cell dysfunction [11, 147]. Inhibition of the CCL2/CCR2 axis reduces tumour infiltration by MDSCs, increases interferon production and stimulates T‐cell responses [148, 149]. However, mixed effects of targeting this axis on tumour progression have been reported [150, 151, 152]. Likewise, VEGF/VEGFR [144, 153] and CXCL12/CXCR4 [154, 155] signalling axes have been targeted to reduce TAM recruitment. Intracellular targets, including p38 MAPK [156] and RNA demethylase ALKBH5 [147, 157], have also been targeted to reduce TAM recruitment.

Active ablation of the TAM compartment also presents treatment possibilities. The bisphosphonate clodronate delivered in a liposomal suspension [158] and trabectedin [142] are toxic to macrophages. The scavenger receptor MARCO has been shown to define immune‐suppressive TAMs in glioblastoma and other cancers, and monoclonal antibodies targeting the MARCO^+^ TAM fraction in carcinomas can enhance tumour immunogenicity and slow growth [159, 160]. Likewise, TAM expression of the scavenger receptor TREM2 is a marker of poor prognosis. GBMs express the highest levels of TREM2 across a panel of cancers [161], and a candidate therapeutic monoclonal antibody, PY314, against TREM2, is available. Nevertheless, effectively delivering large molecules like monoclonal antibodies to the brain compartment remains challenging. Chimeric antigen receptor (CAR) T cells engineered to recognise TAM‐specific surface antigens have also been used to ablate TAM recruitment [162].

### 6.2 TAM compartment re‐education

Tumour‐associated macrophage re‐education has been attempted using diverse strategies in preclinical and clinical settings [104]. Induction of ‘immunogenic’ cancer cell death by conventional chemotherapeutics, such as doxorubicin, can drive TAM activation [163]. Likewise, existing clinically available immunotherapy can re‐educate TAMs, for example, PD‐L1 and PD‐1 checkpoint inhibitors [10, 164, 165] and potentiate the T‐cell cytotoxic effector response for this re‐education. Checkpoint inhibitors have, therefore, served as the basis for several combination re‐education therapy strategies. For example, the conversion of ATP to adenosine by CD73 is associated with the induction of immunosuppressive TAM states. CD73 shRNA knockdown confers survival benefits in combination with checkpoint inhibitors in a preclinical model [166]. An important caveat is the sex‐based differences that may implicate success with checkpoint inhibition and TAM re‐education. Differences in T‐cell exhaustion in males versus females in glioblastoma [167] may warrant careful selection of candidates for immune therapies [75, 168, 169].

The PI3K/mTOR pathway has been targeted to prevent TAM accumulation and to achieve TAM re‐education [24, 170]. Secretion of galectin‐9, associated especially with glioblastoma‐bearing PTEN mutations, drives immunosuppressive polarisation through binding of the Tim‐3 receptor, a key immune checkpoint receptor expressed on TAMs. Checkpoint receptor blockade can impact macrophage polarisation and cytotoxic T‐cell responses, reducing tumour growth in glioblastoma xenografts [171]. Chloroquine, galactan, and Toll‐like receptor agonists have been reported to induce inflammatory myeloid states. At the same time, macrophage activating factors and antibodies directed at CD40 and IL‐1a have also been the subject of trials to achieve macrophage re‐education in solid tumours [172].

Cyclic GMP‐AMP synthase (cGAS) and Stimulator of Interferon Genes (STING) are potent drivers of the innate immune response to pathogens and cancer alike [173]. Mechanistically, cGAS binds pathological cytoplasmic double‐strand DNA associated with tumours and viruses and catalyses cyclic GMP‐AMP (cGAMP) production. In response to this, cGAMP and related pathogen‐associated cyclic dinucleotides bind STING, resulting in the recruitment and activation of Tank‐binding kinase I (TBK1). TBK1, in turn, phosphorylates interferon regulatory factor 3 (IRF3), which translocates to the nucleus and drives type I interferon (IFN) transcription [174, 175]. The cGAS/STING/interferon axis is typically epigenetically silenced in glioma cells, perhaps enabling malignant transformation [176]. Therefore, targeting this axis in the tumour microenvironment, especially TAMs, may be key. The cGAS‐STING/Interferon axis may also be a useful target in TAM‐directed gene therapy, aiming to repolarise the TAM populations of the wider tumour microenvironment by forcing the expression of interferon in these cells, for example [177].

Oncolytic virus infection can simultaneously induce immunogenic malignant cell death and pro‐inflammatory TAM phenotypes in glioblastoma and other cancers [178]. Therefore, brain‐penetrant oncolytic viruses with appropriate tumour selectivity represent a compelling immunotherapy option [179]. However, treatment efficacy will depend on addressing the ability of activated TAMs to restrict virus propagation through the tumour [109, 180].

While these TAM re‐education strategies collectively offer considerable promise, brain cancer is rarely the first choice for treatment development. This reflects the limitations in brain penetration and prediction for antibodies, viral vectors and other large molecules. A further concern relates to specificity and control of treatment response, especially because brain inflammation is very poorly tolerated within the fixed volume of the skull. Therefore, new approaches to achieving efficient and specific delivery to the brain tumour TAM compartment may prove key to delivering on this promise [181].

### 6.3 Gene editing of macrophages

Genetically engineered macrophages (GEMs) can repolarise the tumour microenvironment by expressing pro‐inflammatory cytokines like IL‐21 or by CRISPR knockout at anti‐inflammatory loci like IL‐10 or PD‐L1 [182]. Proof‐of‐principle for this process is typically based on monocytes harvested from peripheral blood. However, editing TAMs *in situ* may ultimately prove possible and desirable, especially as gene delivery to microglia and brain TAMs improves [183]. CAR Macrophages (CAR‐M) represent a special case engineered to recognise tumour‐specific surface antigens. As for CAR‐T cells, the engineered receptors incorporate antigen binding, transmembrane and intracellular signalling domains [184]. Although CAR‐Ms are typically generated by *in vitro* differentiation or *ex vivo* modification of peripheral blood monocytes, CAR‐Ms can also be generated *in situ* by delivering CAR transgenes to TAMs in the wall of the tumour cavity [185]. CAR‐M can phagocytose tumour cells expressing the target antigen and drive adaptive anti‐tumor responses by releasing pro‐inflammatory cytokines and recruitment of cytotoxic effectors, including CD8^+^ T cells [186]. Compared to CAR‐T or CAR‐ Natural Killer (NK) cells, CAR‐M may infiltrate glioblastoma more effectively [187], exploiting the same chemotaxis mechanisms responsible for TAM accumulation. However, optimal surface antigens for CAR‐M targeting remain to be determined. Glioma stem cells typically express CD133 and/or other ‘neural stem cell’ surface markers [181, 188], and the mutant constitutively active EGFRviii receptor is present in many tumours. However, targeting single surface epitopes typically results in the selection and outgrowth of resistant/downregulated clones without a survival benefit [189]. Intravenous infusion is associated with CAR‐M accumulation in liver tissue, impairing treatment effect [190]. CAR‐M may not persist for any prolonged duration following infusion and does not increase effectively *in vivo*. Therefore, effective treatment may depend on repeated CAR‐M dosing. A few clinical trials are underway, but the extent of possible CAR‐M toxicity remains unclear. Additionally, some challenges need to be solved for CAR‐M treatment against glioblastoma. Firstly, the CAR‐M may not exist in patients' bodies for long, even after repeated infusions, which may further limit the effectiveness of the treatment. The second is related to the delivery methods. Lastly, due to the heterogeneity of glioblastoma tissue, the absence of an ideal tumour‐specific antigen represents a bottleneck for CAR‐M application, which is also the plight of CAR‐T therapy against glioblastoma.

### 6.4 Blocking signalling in the TAM compartment

Tumour‐associated macrophage pathway blockade can target specific cell functions and individual interactions mediated through the TAM cell ‘surfactome’ and ‘secretome’. TAMs can promote malignant cell proliferation and invasion in glioma by producing growth factors and signalling moieties, including EGF, TGF‐beta, and stress‐inducible protein 1 [27, 191, 192]. For example, blocking a key TAM‐expressed ‘don't eat me’ signal, such as Clever1, has been associated with reactivating anti‐tumour cytotoxic effector cell responses [193]. Likewise, SIRP1a expressed on TAMs recognises the CD47 antigen, a ‘don't eat me’ signal, upregulated in cancer cells, inhibiting phagocytosis [194, 195]. CD47 antibody blockade, in combination with alkylating chemotherapy, has proved effective in murine preclinical models [196]. Blockade of TAM pro‐angiogenic activity has been most extensively studied in the context of VEGF/VEGFR signalling. While VEGF‐directed monotherapy using bevacizumab has been associated with minimal survival benefit in newly diagnosed glioblastoma [197], combined targeting of VEGF and TIE2 angiogenic growth factor signalling pathways is a promising strategy for targeting the angiogenic TAM state for treatment benefit [198, 199, 200].

## 7 Conclusions

Human glioblastoma myeloid cell states can now be comprehensively profiled through single‐cell transcriptomics, complemented by epigenetic and proteomic techniques. Efforts to collapse the complexity generated by single‐cell transcriptomics into a tractable classification are already bearing fruit, and these will be the basis for better functional understanding and exploitation [44]. The next steps include thoroughly mapping these states to functional phenotypes and manipulation towards improved treatments.

Manipulating the TAM compartment, especially re‐education to induce inflammatory states, remains an appealing but unproven strategy to potentiate tumour response to immunotherapy. However, whereas the biology of glioblastoma malignant cells is well captured in patient‐derived serum‐free cultures [181], the microenvironment has proven much harder to assay *in vitro*. Current TME modelling lacks fidelity or is prohibitively laborious [137], and therefore, accurate and tractable modelling and assay of human myeloid cell states could be transformative for the field. For example, effective capture of human glioblastoma myeloid cell populations *in vitro* could enable stepwise reconstruction and interrogation of the interactions between individual glioma stem cell mutational and transcriptional subtypes and their innate immune microenvironment. Likewise, this approach could support high‐throughput drug and genetic screening approaches.

## Conflict of interest

Harry J. C. J. Bulstrode is funded by a Wellcome Career Development Fellowship. Georgios Solomou is funded by a Cancer Research UK Predoctoral Grant and a Guarantors of the Brain Clinical Research Fellowship. Adam M. H. Young is funded by an NIHR Clinical Lectureship.

## Author contributions

GS and HJCJB conceived, drafted, edited and reviewed the manuscript. AMHY reviewed and drafted the manuscript.
